# Squalamine: An Appropriate Strategy against the Emergence of Multidrug Resistant Gram-Negative Bacteria?

**DOI:** 10.1371/journal.pone.0002765

**Published:** 2008-07-23

**Authors:** Chanaz Salmi, Celine Loncle, Nicolas Vidal, Yves Letourneux, Jacques Fantini, Marc Maresca, Nadira Taïeb, Jean-Marie Pagès, Jean Michel Brunel

**Affiliations:** 1 Unité de Recherche sur les Maladies Infectieuses et Tropicales Émergentes (URMITE) UMR 6236, Faculté de Médecine, Université de la Méditerranée, Marseille, France; 2 Laboratoire Interactions Moléculaires et Systèmes Membranaires (IMSM), CNRS UMR 6231, CRN2M, Faculté des Sciences de St-Jérôme, Université Paul Cézanne, Marseille, France; 3 UMR-MD1, Facultés de Médecine et de Pharmacie, Marseille, France; The Scripps Research Institute, United States of America

## Abstract

We reported that squalamine is a membrane-active molecule that targets the membrane integrity as demonstrated by the ATP release and dye entry. In this context, its activity may depend on the membrane lipid composition. This molecule shows a preserved activity against bacterial pathogens presenting a noticeable multi-resistance phenotype against antibiotics such as polymyxin B. In this context and because of its structure, action and its relative insensitivity to efflux resistance mechanisms, we have demonstrated that squalamine appears as an alternate way to combat MDR pathogens and by pass the gap regarding the failure of new active antibacterial molecules.

## Introduction

**Figure 1:**
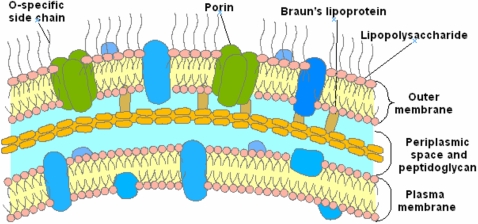
Gram negative bacteria envelope.

**Figure 2:**
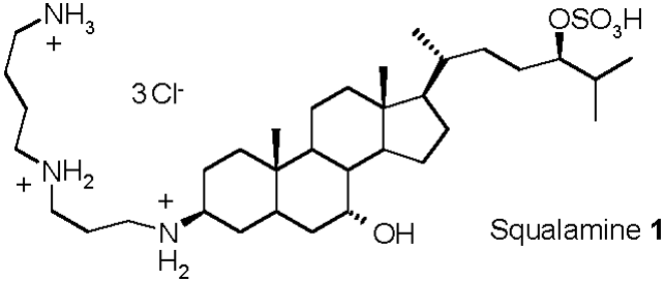
Structure of squalamine 1.

The emergence of multidrug resistant bacteria/pathogens has highlighted the need for the development of new antibiotics.[1]–[3] In this area, drug resistance of Gram-positive organisms has received significant attention with respect to Gram-negative bacteria which are innately resistant to many common antibiotics due to their envelope structure. In Gram-positive and Gram-negative bacteria, resistance to membrane active antibiotics requires major changes in membrane organization, which in turn influence the permeability barrier increasing susceptibility to hydrophobic antibiotics. The outer membrane of Gram-negative bacteria forms an effective barrier to such molecules.[4] Consequently, numerous antibiotics that are active against Gram-positive organisms are much less active against Gram-negative bacteria. In the latter case, the outer membrane contains lipopolysaccharide (LPS) which creates the asymmetry of the membrane architecture (Figure 1).[5]–[7] It is widely held[8] that the permeability barrier of the outer membrane is increased via cross-bridging between LPS and divalent cations.[9], [10] Thus, metal ion chelators such as EDTA, certain cationic antimicrobial peptides[11]–[13] and polyamines[14]–[16], which can alter the binding of divalent cations, are able to disrupt the organization of the outer membrane, increasing its permeability, and therefore sensitise bacteria to hydrophobic antibiotics. In this context, an attractive approach for the development of antibacterial agents is the use of compounds targeting outer membranes of Gram-negative bacteria since they are not expected to readily induce resistance formation. In recent years, a wide variety of low molecular weight antibiotics including peptides, lipids and alkaloids have been isolated from diverse animal species.[11], [12], [17]–[19] Among these substances, a water soluble cationic amino sterol namely squalamine **1** (7,24-dihydroxylated-24 sulfated cholestane conjugated to spermidine group at C-3) has been isolated from the dogfish shark *Squalus acanthias* (Figure 2). This compound exhibits potent antimicrobial activity and high minimum haemolytic concentration (>200 µg/mL) suggesting its potential application in human medicine.[20]–[24] We will report on the the broad spectrum of antibacterial activity of squalamine against sensitive and resistant bacterial strains. We also demonstrate its mechanism of action towards Gram-negative bacteria suggesting that this molecule constitutes one of the most appropriate responses against the questionable emergence of multidrug resistant Gram-negative bacteria and associated nosocomial diseases.

## Results and Discussion

Our first study concerning the antimicrobial activities of squalamine **1** demonstrated its efficiency towards fungal and bacterial strains with Minimum Inhibitory Concentrations (MIC) varying from 2.5 to 25 µg/mL (Table 1–2). It is also noteworthy that similar activities have been demonstrated against sensitive and resistant Gram-negative bacteria (*Escherichia coli* and *Pseudomonas aeruginosa*). The re-use of “old” drugs such as polymyxins has been proposed as an alternative or rescue therapy for patient infected by MDR strains.[25], [26] We have recently reported that two clinical *Enterobacter aerogenes* isolates have developed resistance to polymyxins involving an alteration of LPS after colistin was used during the therapy. This modification did not alter the protein profile of outer membrane.[27] The first isolate, strain C (Table 3) presenting a polymyxin B susceptibility was sensitive to low concentrations of squalamine **1**. Interestingly, clinical isolates D and E that presented a high level of polymyxin resistance (32-fold increase of MIC) exhibited a decrease of squalamine susceptibility with a five-fold increase of the corresponding MIC. This result suggested that the alterations of LPS previously reported in these isolates and causing the resistance towards polymyxin B[27], are able to modulate the squalamine activity. In this context, regarding the other antibiotic families, squalamine offers advantages associated with its activity properties. The squalamine action is preserved even in MDR clinical isolates that overexpress various mechanisms of resistance including drug efflux pumps, alteration of membrane permeability caused by absence of porins, enzymatic barrier, all well-known mechanisms which induce high level of resistance towards quinolones, ß-lactams, phenicols, etc (Table 1–3). For instance: (i) strain 289 was completely devoid of porins, expressed high level of AcrAB-Tol C efflux and a simultaneous overproduction of β-lactamase activity, (ii) strain 298 (289 derivative) exhibited the same phenotype but was deleted of Tol C efflux component, (iii) strain C was porin defficient, overexpressed AcrAB-Tol C efflux and exhibited a lipopolysaccharide (LPS) wild type profile, (iv) strains D and E had same phenotype plus LPS modifications.[27], [28] Thus, the activity of squalamine **1** suggests in a first approach that its biological activity results from the synergistic combination of an anionic bile salt with spermidine, each of which independently exhibit considerably less antibiotic activity than squalamine **1**.[29], [30]

**Table 1:** Antimicrobial activities of squalamine **1**.

Sensitive Strain	MIC, µg/mL							
	*S. cerevisiae* (CIP 28383)	*C. albicans* (CIP 1180-79)	*S. aureus* (CIP 4.83)	*E. faecalis* (CIP 103015)	*E. hirae* (ATCC 10541)	*E. coli* (ATCC 54127)	*P. aeruginosa* (ATCC 15442)	*E. aerogenes* (ATCC 15038)
**Squalamine**	25	>20	3.12	12.5	10	2.5	8	20

**Table 2 pone-0002765-t002:** Antimicrobial activities of squalamine **1** towards bacteria resistant strains.

Bacteria resistant strains	MIC (µg/mL)
*E. coli* AG100	2
*E. coli* AG100A	1
*E. coli* AG100Atet	2
*P. aeruginosa* PA01	16
*P. aeruginosa* Z61	4
*P. aeruginosa* 124	16

**Table 3:** Antibacterial susceptibility of various Multidrug resistant (MDR) *E. aerogenes* clinical isolates expressing various antibiotic resistance mechanisms.

*E. aerogenes* strains	MIC (µg/mL)				
	Norfloxacin	Chloramphecol	Cefepime	Polymyxin B	Squalamine
289, MDR isolate	256	512	64	nd	**4**
298, 289 *tolC*- derivative	16	32	64	nd	**2**
C, MDR isolate	256	16	32	0.25	**2.5**
D, MDR isolate	128	16	8	16	**10**
E, MDR isolate	128	16	8	32	**12.5**

Even if strong antibacterial activities have been noticed, the mechanism of action of squalamine towards Gram-negative bacteria remains questionable. Thus, two possible modes of action for such an antibacterial molecule can be underlined (i) competitive binding to a cell-surface exposed receptor (*e.g.* such as porin)[6], [10] involved in key cellular processes and (ii) channel or pore formation in the cytoplasmic membrane. Recently, Katsu *et al.* examined the structure-activity relationship between original polyamines (naphthylacetylspermine and methoctramine) and the outer membrane of Gram-negative bacteria demonstrating that lipophilic moieties and a number of amino groups in polyamines were important to permeabilisation.[31]

**Figure 3:**
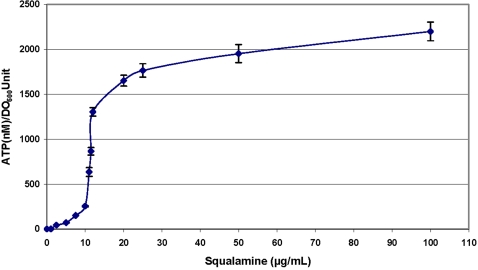
Measurement of squalamine concentration effect on *E. coli* ATP efflux.

**Figure 4:**
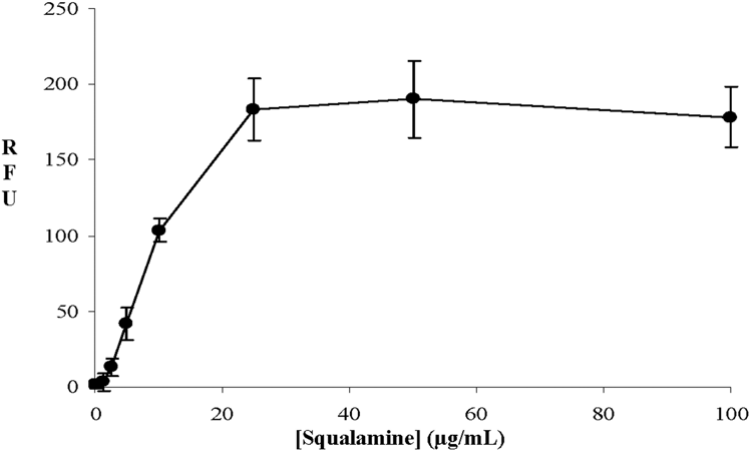
Effect of squalamine on bacterial membrane integrity assessed by fluorescence measurement of propidium iodide – DNA/RNA interactions. Results are expressed in relative fluorescence unit (RFU) as Mean±S.D. (n = 3, three independent experiments).

**Figure 5:**
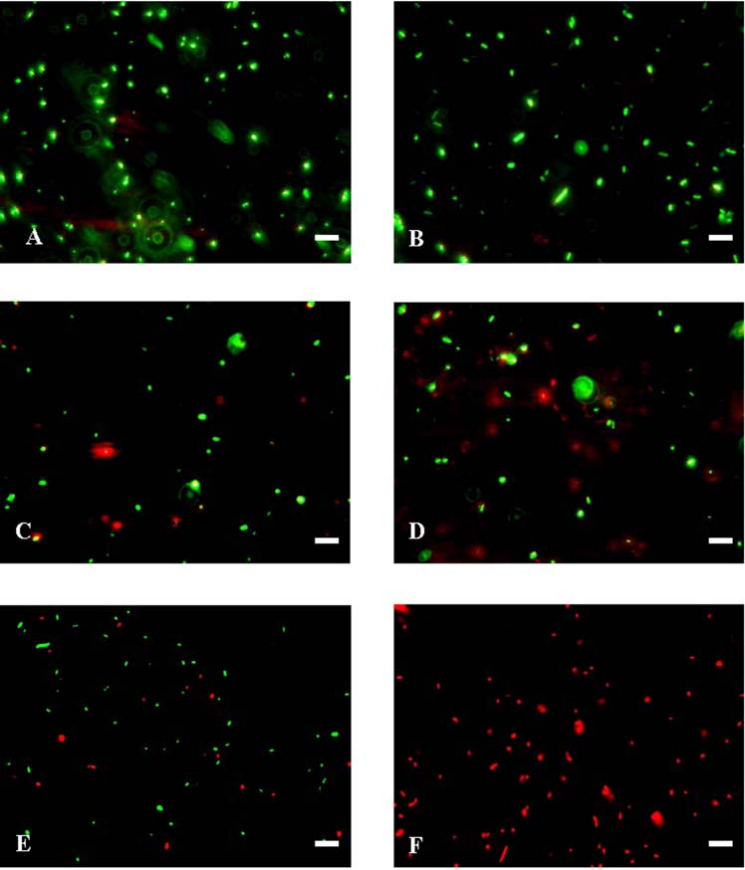
Fluorescence-based microscopic evaluation of the effect of squalamine on bacterial integrity and survival. *E. Coli* cultures (10^9^/mL) were either left untreated (A) or treated with increasing squalamine concentrations: 1.25 µg/mL (B), 2.5 µg/mL (C), 5 µg/mL (D), 10 µg/mL (E), 100 µg/ml (F). Suspensions of cultured *E. Coli* were then stained with the Live/Dead BacLight bacterial Viability kit, as described in material and methods. Scale bar (10 µm).

A bioluminescence method was used to determine the effect of squalamine addition on the intracellular pool of bacterial ATP. The detection of external concentration of ATP was then used as a reporter reflecting the permeabilizing effect of squalamine along with the dose-effect relationships. Thus, it clearly appears that for a squalamine concentration of about 20 µg/mL, 80% of the intracellular ATP has been released in the medium suggesting the disruption of the membrane barrier (Figure 3). In addition to ATP release measurements, effect of squalamine on bacterial membrane integrity was also assessed using the cell-impermeable DNA/RNA dye propidium iodide (PI) (Figure 4). Results showed that squalamine caused a dose-dependent increase in PI-associated fluorescence. At a 1.25 µg/mL concentration, squalamine did not significantly affect PI-associated fluorescence (2±3.5-fold increase, *p* = 0.42). Increase only started to be significant at 2.5 µg/mL (8.0±3.4 fold increase (*p*<0.05)) and was maximal at 25 µg/mL squalamine concentration (110.0±12.5-fold (*p*<0.001)). Similarly, CTAB known to cause bacterial permeabilisation, induced equivalent increases in PI-associated fluorescence 100.0±11.5-fold increase compared to vehicle-treated bacteria, *p*<0.001). Finally, in order to investigate membrane alterations associated with squalamine action, we have used an assay based on fluorescent microscopy (Live/Dead BacLight, Molecular Probes) (Figure 5). In that assay, live bacteria appear green whereas dead bacteria with an alteration of their membrane permeability are red. At 0 or 1.25 µg/mL of squalamine, all bacteria appeared green (Figure 5 A and B). Red/dead bacteria started to be observed with concentration of squalamine higher than 2.5 µg/mL (Figure 5, C). Moreover, the cytotoxicity increased when bacteria colonies are treated with a higher squalamine concentration (i.e. 100 µg/mL), under this condition, all the bacteria were stained red (Figure 5, F) in contrast to the untreated ones fluorescing green.

On the other hand, it is well known that the effects of chaotropic agents can be impaired by exogenous divalent cationic ions (Mg^2+^ or Ca^2+^).[5] In this context, the effect of various monovalent or divalent ions on bactericidal activity of squalamine **1** has been investigated using 1 mM concentration salts. As shown in Table 4 the addition of monovalent salts did not block the activity of squalamine towards *E. coli* whereas this later was completely abolished by the same concentration of divalent ions such as MgCl_2_ or CaCl_2_. Regarding these divalent salts, it was demonstrated that the full activity of squalamine is obtained at low concentrations i.e. approximately 0.09 mM.

**Table 4:** Effects of various monovalent or divalent salt solutions (1 mM) on bactericidal activity of squalamine (2.5 µg/mL).

Salts [1 mM]	Inhibition effect^a^
No salt	NI
NaCl	NI
NaBr	NI
NaI	NI
KCl	NI
KBr	NI
CaCl_2_	++
MgBr_2_	++
MgCl_2_	++

a NI: No inhibition; ++: Total inhibition (100% Bacterial survival).

**Figure 6:**
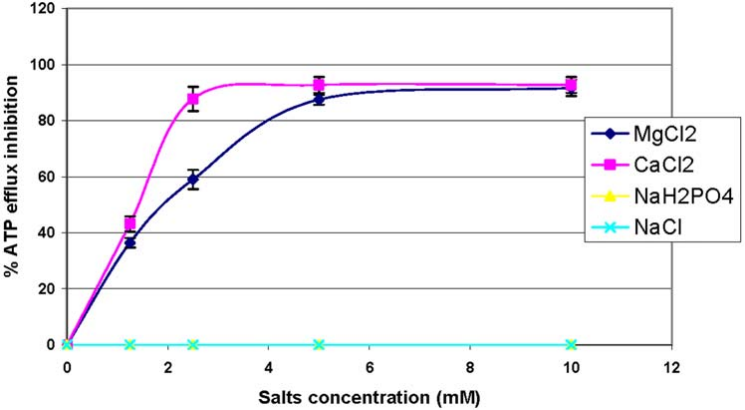
ATP efflux inhibition in *E. coli* in the presence of squalamine (5 µg/mL) and various mono and divalent salt solutions.

Moreover, using bioluminescence method, a noticeable inhibition of the *E. coli* ATP efflux squalamine-dependent was observed in the presence of divalent ions at various concentrations after 10 minutes of incubation (Figure 6). Thus, NaCl and NaH_2_PO_4_ did not lead to any inhibition on *E. coli* ATP efflux in the presence of squalamine whereas a dramatic inhibition of this efflux was observed in the presence of CaCl_2_ or MgCl_2_. Moreover, a total inhibition of the ATP efflux was reached by a concentration of about 5 mM or 2.5 mM for MgCl_2_ or CaCl_2_, respectively.

Many groups have observed the antibacterial effect of cationic surfaces on Gram-positive as well as Gram-negative bacteria. This suggests that the mechanism is not system-specific, contrary to that which is generally the case with antibiotics. We surmise, as already hinted by others[32], that the death process involves electrostatic interactions and is related to the high density of charges exposed at the surface of bacterial membranes. The architecture of LPS, the main component of outer leaflet of the outer membrane, favors the presence of a large number of negative charges that may stimulate the interactions with cationic substrates.[6], [33] The role of LPS is partially suggested with the modulation of squalamine activity in polymyxin resistant isolates and (Table 3).

To propose the existence of a charge-density threshold in the squalamine mode of action, we are helped by recent advances in the understanding of the electrostatic interactions between polyelectrolyte chains and oppositely charged surfaces.[32] It has been gradually realized that adsorption in such cases is driven by the release in solution of the counterions initially confined within the respective electrical double layers. The same process applies to bacteria, which can be crudely considered as large two dimensional polyelectrolytes. Upon adsorption on a cationic solid substrate, the electrostatic compensation of the negative charges of the bacterial envelope is provided by the cationic charges of the substrate, and the bacteria lose their natural counterions.

As previously outlined, squalamine is an amphipathic compound which interacts with various membrane glycerophospholipids with distinct affinities.[34] As phosphatidylglycerol is the main glycerophospholipid in bacterial membranes whereas phosphatidylcholine is more abundant in eukaryotic membranes, this may explain why squalamine could kill bacteria more easily than mammalian cells. Nevertheless, although Gram-positive bacteria have a single membrane that is enriched in phosphatidylglycerol, Gram-negative bacteria also have an external membrane in which the predominant lipid is lipopolysaccharide (LPS). LPS is the major glycolipid recovered from a Folch extract of Gram-negative *E. coli* bacteria. To study the potential interaction with squalamine and a reconstituted bacterial membrane containing LPS, a lipid extract of *E. coli* enriched in LPS was spread at the air-water interface where it formed a stable lipid monolayer. Squalamine was then added in the aqueous subphase and its insertion within the LPS film was assessed by surface pressure measurements[35] As shown in Table 5, squalamine penetrated the LPS monolayer at concentrations as low as 0.5 µg/mL. In contrast, higher doses of squalamine were necessary to allow its insertion in monolayers consisting of either neutral glycosphingolipids or gangliosides extracted from lymphocytes. Thus, as far as glycolipids are concerned in early squalamine-membrane interactions, it is clear that bacterial LPS is significantly more active than eucaryotic glycolipids. Squalamine also interacted with matured lipid A (the membrane-anchored backbone of LPS), in a divalent cation-dependent way. Indeed, this squalamine-membrane interaction is highly cationic divalent ion dependent which is consistent with the previously demonstrated lack of activity of squalamine in the presence of such ions in the medium. Moreover, squalamine interacted very poorly with GalCer, but very actively with ceramide (Cer), the membrane-anchored backbone of sphingolipids. This may suggest that the insertion of squalamine into eukaryotic membranes could be impaired by the sugar part of glycolipids. Overall these data provide a biochemical basis for the potent activity of squalamine on bacterial Gram-negative and Gram-positive membranes and its relative lack of activity on eukaryotic membranes. Further physicochemical studies will be conducted in the near future in order to decipher the molecular mechanisms (including divalent cation dependence) controlling this striking lipid selectivity.

**Table 5:** Measurements of squalamine interactions with various bacterial and eukaryotic lipids.

Squalamine concentration (µg/mL)	*E. coli* Extract (LPS)	Lymphocyte Neutral GSL	Lymphocyte Gangliosides	Lipid A	Lipid A 1 mM MgCl_2_	GalCer	Cer	GM1	GD3	GT1b
**0.5**	**+**	**−**	**−**	**−**	**−**	**−**	**+**	**−**	**−**	**−**
**1.0**	**+**	**−**	**−**	**−**	**−**	**−**	**+**	**−**	**−**	**−**
**1.5**	**+**	**+**	**−**	**+**	**−**	**−**	**+**	**−**	**+**	**−**
**2.0**	**+**	**+**	**−**	**+**	**−**	**−**	**+**	**+**	**+**	**−**
**≥2.5**	**+**	**+**	**+**	**+**	**+**	**+**	**+**	**+**	**+**	**+**

### Conclusion

Squalamine is a membrane-active molecule that targets the membrane integrity as demonstrated by the ATP release and dye entry. Consequently, its activity may depend on the membrane lipid composition. It is worthwhile mentioning that the alteration of LPS involved in the polymyxin-resistant clinical isolates moderately changes the squalamine MIC preserving the activity spectrum of the molecule compared to polymyxin B. Thus, if we consider that squalamine acts as a “membranotropic” molecule, it remains possible to observe less susceptible strains like those isolated after polymyxin treatment. However, the resistant variants must preserve a sensitivity level since the adaptation stress requires strong changes in membrane structure which drastically deal with intrinsic membrane stability and the bacterial fitness. In addition, this molecule shows a preserved activity against bacterial pathogens presenting a noticeable MDR phenotype concerning usual antibiotics. Squalamine has membranotropic properties regarding its bacterial membrane activity and due to its structure containing a cholestanol core it exhibits a moderate level of side effect on eukaryotic cells at doses that kill MDR bacterial pathogens. In this context and because of its structure, action and its relative insensitivity to efflux resistance mechanisms, squalamine may be an alternate way to combat MDR pathogens and by pass the gap regarding the failure of new active antibacterial molecules. This aspect is especially important since some recently described molecules having an active antibacterial spectrum are also substrates for efflux pump systems resulting in a decrease of activity in MDR strains, *e.g.* peptide deformylase inhibitor, plectasin, platensimycin.[36]–[38]

## Materials and Methods

### Determination of minimal inhibitory concentrations

Antimicrobial activity of the compounds was studied by determination of minimal inhibitory concentrations (MIC) according to the NCCLS guidelines M7-A2 using the microbroth dilution methods. All the reference strains were issued from the Institut Pasteur collection (Paris). The other reference strains and clinical isolates were from the UMR-MD1 collection and have been previously described.[27], [28], [39] The bacteria strains were grown on trypticase soy agar (Becton Dickinson) at 37°C in LB or MH broth for *E. coli*, *E. aerogenes and S. aureus* or BHI broth for *E. faecalis*. Inocula were prepared in the respective medium by ajusting the cell density.

Antimicrobial activities of the compounds were determined by using a broth microdilution method performed in sterile 96-well microplates. The molecules were diluted in water and were transferred to each microplate well in order to obtain a two-fold serial dilution and inocullum containing 2–6 10^5^ CFU of each bacteria was added to each well. A number of wells were used for positive controls, inoculum viability and solvent effect. Results were read after 18 hours at 37°C and the MIC was the lowest concentration of the antibacterial agent at which no growth was detected. MIC values are the mean of three independent experiments.

### Measurement of ATP efflux

Squalamine solutions were prepared in doubly distilled water at different concentrations. A suspension of growing *E.coli* to be studied in LB broth was prepared and incubated at 37°C. 90 µL of this suspension was added to 10 µL of squalamine solution and vortexed for 1 second. 50 µL of luciferin-luciferase reagent (Yelen, France) was immediately added to the precedent mix and luminescent signal quantified with a Lucy luminometer (Yelen, France) for five seconds. ATP concentration was quantified by internal sample addition.

### Measurement of ATP efflux inhibition in *E. coli* in the presence of squalamine (25 µg/mL) and various mono and divalent salt solutions

1 M salts (CaCl_2_, MgCl_2_, NaH_2_PO_4_, NaCl) solutions were prepared in doubly distilled water and diluted in a 250 µg/mL squalamine solution for 10 minutes at room temperature. Then, ATP efflux was measured with the protocol described above. Results were expressed as ATP efflux percent inhibition relative to the salt free squalamine solution.

### Membrane permeability assessment

Over night bacterial suspensions of *E. coli* in LB were centrifuged 10 min at 10 000 g. Bacterial pellets were resuspended in PBS at 2.5×10^9^ bacteria per mL, as bacterial permeabilisation assays giving identical results in PBS or LB. Bacteria were added into 96-well black NUNC plate with 0.5×10^9^ bacteria added per well. The cell-impermeable DNA/RNA dye propidium iodide (PI, Sigma) was then added to bacteria at a final concentration of 30 µM. After 10 min of equilibration at 37°C, bacteria were treated with dye alone or with increasing concentrations of squalamine; CTAB at 58 µg/mL (i.e. 160 µM)[40] being used as positive control of membrane permeabilisation. Finally, fluorescence was measured after 30 min of incubation at 37°C using a microplate Fluoroscan Ascent spectrofluorometer (excitation at 540 nm and emission at 590 nm), as preliminary experiments have shown that maximal fluorescence was obtained at that time independently of the dose of squalamine.

### Fluorescence microscopy

In order to investigate membrane damage, we have used the fluorescence based Live/Dead BacLight assay (Molecular Probes). This assay contains a mixture of two nucleic acid stains: a green-fluorescent Stylo 9 stain and a red-fluorescent propidium iodide stain. These stains differ to their ability to penetrate healthy bacterial cells. Intact cell membranes stains green, whereas bacteria with damaged membranes stains red. Live and dead bacteria were viewed simultaneously by fluorescence microscopy with suitable optical filter sets. *Escherichia coli* cells (10^9^ CFU/mL) were incubated in the presence of different concentrations of squalamine (1.25 µg/mL, 2.5 µg/mL, 5.0 µg/mL, 10 µg/mL, 100 µg/mL) for 30 minutes at 37°C. Suspensions of treated and untreated cells were stained according to BacLight assay instruction and 5 µL of each bacterial suspensions were subsequently deposited on slides and analyzed using a fluorescence microscope.

### Measurements of squalamine interactions with various bacterial and eucaryotic lipids

The lipids were spread at the air-water interface at an initial surface pressure of 15 mN.m^−1^. After evaporation of the solvent (hexane/chloroform/ethanol; 11∶5∶4, vol∶vol∶vol), squalamine was injected in the pure aqueous subphase (volume 800 µL). The variations of the surface pressure were continuously recorded with a fully automated microtensiometer (μTROUGH SX, Kibron Inc. Helsinki, Finland). All experiments were carried out in a controlled atmosphere at 20°C±1°C. The data were analyzed with the Filmware 2.5 program (Kibron Inc. Helsinki, Finland). The accuracy of the system under our experimental conditions was ±0.25 mN.m^−1^ for surface pressure. A plus means that the surface pressure increase was above 5 mN.m^−1^ after 60 minutes of interaction, a minus means that during the same period of time, the surface pressure increase did not exceed 2 mN.m^−1^. Bacterial and eukaryotic lipids were extracted and submitted to a Folch partition as described previously.[41] Lipopolysaccharide (LPS) was the only glycolipid recovered from the Folch lower phase of the bacterial extract, as demonstrated by high performance thin layer chromatography. The Folch lower phase of lymphocyte lipids contained GlcCer, LacCer, Gb3 and Gb4. The upper phase contained GM1, GM3 and GD3. Pure Lipid A, Cer, GalCer, GM1, GD3 and GT1b were purchased from Sigma. Each experiment was performed three times with similar results.
